# Chitosan Resin-Modified Glass Ionomer Cement Containing Epidermal Growth Factor Promotes Pulp Cell Proliferation with a Minimum Effect on Fluoride and Aluminum Release

**DOI:** 10.3390/polym15173511

**Published:** 2023-08-23

**Authors:** Chanothai Hengtrakool, Supreya Wanichpakorn, Ureporn Kedjarune-Leggat

**Affiliations:** 1 Department of Conservative Dentistry, Faculty of Dentistry, Prince of Songkla University, Hat Yai, Songkhla 90112, Thailand; htkchino@gmail.com; 2 Department of Oral Biology and Occlusion, Faculty of Dentistry, Prince of Songkla University, Hat Yai, Songkhla 90112, Thailand; supreya.w@psu.ac.th; 3 Cell Biology and Biomaterials Research Unit, Faculty of Dentistry, Prince of Songkla University, Hat Yai, Songkhla 90112, Thailand

**Keywords:** chitosan resin-modified glass ionomer cement, EGF, fluoride, aluminum, chitosan

## Abstract

The development of biomaterials that are able to control the release of bioactive molecules is a challenging task for regenerative dentistry. This study aimed to enhance resin-modified glass ionomer cement (RMGIC) for the release of epidermal growth factor (EGF). This RMGIC was formulated from RMGIC powder supplemented with 15% (*w*/*w*) chitosan at a molecular weight of either 62 or 545 kDa with 5% bovine serum albumin mixed with the same liquid component as the Vitrebond. EGF was added while mixing. ELISA was used to determine EGF release from the specimen immersed in phosphate-buffered saline at 1 h, 3 h, 24 h, 3 d, 1 wk, 2 wks, and 3 wks. Fluoride and aluminum release at 1, 3, 5, and 7 d was measured by electrode and inductively coupled plasma optical emission spectrometry. Pulp cell viability was examined through MTT assays and the counting of cell numbers using a Coulter counter. The RMGIC with 65 kDa chitosan is able to prolong the release of EGF for significantly longer than RMGIC for at least 3 wks due to its retained bioactivity in promoting pulp cell proliferation. This modified RMGIC can prolong the release of fluoride, with a small amount of aluminum also released for a limited time. This biomaterial could be useful in regenerating pulp–dentin complexes.

## 1. Introduction

Growth factors and active biological molecules play a crucial role in the successful regeneration and repair of dentin–pulp complexes [1,2]. The development of a possible new generation of biomaterials to control the release of bioactive molecules is, therefore, challenging work.

Glass ionomer cement (GIC) is an acid-base cement produced from the reaction of fluoro-aluminosilicate glass powder with poly(acrylic acid) and is widely used in dental and medical applications due to its biocompatibility, antibacterial properties, sealing ability, and capacity to prolong the release of fluoride. There have been some attempts to enhance the sustained release of substances from this cement [3], such as CPP-ACP [4], chlorhexidine [5], and surface pre-reacted glass ionomer (S-PRG) filler [6], especially in terms of protein [7]. Our group discovered that chitosan-fluoro-aluminosilicate glass ionomer cement could prolong the release of bovine serum albumin (BSA) without alteration of its molecular weight, and this cement does not increase toxicity toward pulp cells [7]. BSA was used as the released protein because of its good biocompatibility with cells and its function as a carrier protein, thus helping the release of other small proteins [8]. This material can prolong the release of protein, possibly due to the formation of a polyelectrolyte complex between the cationic group of chitosan and the anionic group of poly(acrylic acid) [9]. Chitosan is a biocompatible, biodegradable, natural biopolymer that is a copolymer of glucosamine and N-acetylglucosamine derived from chitin. Chitosan has been used widely in biomedical areas, such as bone scaffolds, tissue engineering, and controlled drug or biological molecule release [10]. This polymer can form an insoluble complex from the reaction of chitosan and polyacrylic acid, and this complex has been proven as a controlled drug delivery system [11,12]. Some studies have reported the antibacterial properties of chitosan-modified GIC cement [13,14]. Recently, chitosan in various forms has been studied to improve some properties of GIC, such as increasing osteogenic potential in osteosarcoma cells [15] and adding quaternized chitosan-coated mesoporous silica nanoparticles in order to improve mechanical properties, increase fluoride release, and enhance antibacterial effects [16].

Resin-modified glass-ionomer cement (RMGIC), or light-cured GIC, is the development of glass ionomer cement by adding resin, especially 2-hydroxyethyl methacrylate (HEMA), in order to control the setting of the cement by light-activated polymerization, which allows the clinician more time to work with the material. The main components of RMGIC are similar to conventional GIC, namely fluoro-aluminosilicate glass powder and poly(acrylic acid). Thus, it is feasible for modified RMGIC to control the release of growth factors through chitosan. A previous study revealed that chitosan-modified RMGIC supplemented with translationally controlled tumor protein, an anti-apoptotic protein, can reduce the cytotoxicity of residual HEMA from this RMGIC [17]. This study aimed to modify RMGIC with two molecular weights of chitosan and albumin in order to prolong the release of the growth factor. The growth factor that was used in this study was human epidermal growth factor (EGF). It is a low molecular weight protein (about 6.3 kDa) and has a mitogenic or cell proliferation property [18]. A recent study reported that EGF can also induce neural differentiation in dental pulp stem cells [19]. The important properties of the glass ionomer cement, especially fluoride and aluminum release, were also investigated.

## 2. Materials and Methods

### 2.1. Materials

The RMGIC used in this study was the Light Cure Glass Ionomer Liner/Base (Vitrebond TM) from 3M ESPE (3M, ESPE, St. Paul., MN, USA). The RMGIC powder was composed of fluoro-aluminum-zinc-silicate glass (batch no. 7LB2010-05 for the study of EGF release and batch no. 7 ME 2010-10 for Al and F measurements), and the liquid (batch no. 7HJ2010-02 and 7HP2010-04) was composed of 40% itaconacid-isocyanoethyl-methacrylate acrylic acid, 24% HEMA, and water. Chitosan was used at two molecular weights (Mw): 62 kDa, degree of deacetylation (DD) = 89% (Ta Ming Enterprises, Samutsakorn, Thailand), and 545 kDa, DD = 79% (Fluka, Steinheim, Switzerland, batch no. 50494). Cell culture medium and supplements were products of Gibco (Invitrogen Corporation, Grand Island, NY, USA). EGF was sourced from Promega (Promega, Madison, WI, USA).

### 2.2. Specimen Preparation

Two different diameters of specimens (but with the same 1 mm thickness) were used here. One was a diameter of 10 mm, which was used for the study of EGF, fluoride, and aluminum release, and another was 5 mm, which was used for cell culture experiments.

The powder of the innovative RMGIC was composed of the RMGIC powder mixed with 5% by weight bovine albumin and 15% by weight chitosan. There were two groups of the innovative RMGIC according to the type of chitosan: the first group using chitosan at an Mw of 545 kDa, referred to as the GI+C(F) group, and the second group using chitosan at a lower Mw of 62 kDa, referred to as GI+C(K). The liquid part consisted of the same components as the commercial resin-modified glass ionomer cement, representing a control group (GI), as described above. The composition of the powder in the groups of different RMGICs is shown in Table 1.

The types of cement were dispensed according to the manufacturer’s instruction of RMGIC, with a 1.4:1 powder:liquid weight ratio. The EGF release groups were supplemented with 4 or 8 µg/mL of EGF in 1% Albumin in PBS, which was added during mixing the powder and the liquid part in order to have about 40 ng of total EGF for each specimen. The mixed cement was hand spatulated to form a uniform mix and transferred into ring Teflon molds. Polythene sheets and glass slides were then placed over the filled mold, after which light hand pressure was applied. Then, the cement was photocured for 40 s on both sides of the mold with curing light (sds Kerr, LE Dementron, Kerr Corporation, Danbury, CT, USA) at a wavelength of 450–470 nm. Specimens were retained in the molds for 1 h during storage in an incubator at 37 °C. This procedure was to complete the maturation of the material prior to further investigations.

### 2.3. Determination of EGF Releasing

There were three groups of the RMGICs, GI+EGF, GI+C (F) +EGF, and GI+C(K)+EGF. Each group had 6 specimens. After specimen storage in an incubator, they were removed from their molds, and then each specimen was weighed to an accuracy of 0.0001 g using a digital balance (Sartorius MC210, Goettingen, Germany). After weighing, each specimen was stored at 37 °C in individual pots containing 1 mL of phosphate-buffered saline (PBS) of pH 7.4 with 1 mM of phenylmethanesulfonyl-fluoride, which is a proteinase inhibitor for preventing EGF degradation. The storage pot was continually shaken at low speed (50 rpm) in an incubator at 37 °C. All storage solutions were replaced with a similar volume of fresh PBS at 1 h, 3 h, 24 h, 3 days, 1 week, 2 weeks, and 3 weeks, respectively. The amount of the released EGF was determined using the sandwich ELISA technique. High-binding ELISA plates (Nunc, Roskilde, DK) were coated with 5 µg/mL of monoclonal anti-human EGF antibody in 100 µL of phosphate-buffered saline (PBS) per well (R&D Systems, Minneapolis, MN, USA) for 24 h. The liquid was removed and rinsed twice with 300 µL/well of wash buffer (0.05% Tween 20 in PBS pH 7.4) using a Titertek Microplate Washer (Flow Laboratories, Lugano, Switzerland) before each new addition. The plates were blocked with 300 µL/well of PBS, which contained 1% BSA, 5% sucrose, and 0.05% NaN3, and were incubated at room temperature for 1 h before washing. Then, 100 µL of each sample, including standard EGF (Promega, Madison, WI, USA), at a concentration between 5 and 200 pg/mL, was diluted with diluent and incubated for 2 h at room temperature. After washing, the plates were left with 100 µL/well of 50 µg/mL of biotinylated anti-human EGF antibody and left for 20 min before washing. Then, 100 µL/well of streptavidin HRP (R&D Systems) diluted at 1:200 with PBS containing 1% BSA was added and incubated at room temperature for 20 min. Following this, 100 µL/well of substrate solution (1:1 of Color reagent A, H2O2, and Color reagent B, Tetramethylbenzidine, R&D Systems) was added and left in a dark place for 20 min before stopping the reaction with 1 M H2SO4 and reading the optical density immediately by a microplate reader (Titertek Multiskan, Flow Laboratories, Lugano, Switzerland), using dual-wavelength at 450 and 570 nm. The optical density was calculated by subtracting the readings at 570 nm from the readings at 450 nm. The data was linearized using log transformation of absorbance and concentration of standards, and then the EGF of samples was calculated using regression analysis.

### 2.4. Fluoride and Aluminum Release Measurement

Specimens were allocated to one of 6 groups, as shown in Table 1. Each group was composed of 6 specimens. After the removal of the specimens from the molds, the excess material was expelled. The specimens were weighed using a digital balance (±0.0001 g), and the dimensions were measured in order to confirm the same size of each specimen prior to immersion in each plastic container containing 10 mL of deionized water. The sealed plastic container was continually shaken at low speed (50 rpm) in an incubator at 37 °C. All storage solution was replaced with a similar volume of fresh deionized water commencing at 1, 3, 5, and 7 days.

#### 2.4.1. Fluoride Analysis

The fluoride concentration of the storage solution was measured using an ion-selective electrode system consisting of an ion analyzer (Expandable ion analyzer EA 940, Orion Research, Cambridge, MA, USA) and a combination fluoride electrode (Orion Research, Cambridge, MA, USA). The measurement solution was performed by mixing 0.5 mL of each sample solution with 1.5 mL of 0.1 M hydrochloric acid in a plastic container [20]. Standard solutions with fluoride ranging from 1 to 100 ppm were used to calibrate the system prior to sample measurement and recalibrated every 1 h to compensate for local temperature and humidity changes. Three readings of fluoride concentrations from each solution were recorded in parts per million. The amount of fluoride was calculated as milligrams of fluoride released per gram of glass-ionomer cement (mg F/g cement).

#### 2.4.2. Aluminum Analysis

The aluminum concentration of the storage solution was measured using an inductively coupled plasma-optical emission spectrometer, ICP-OES (Perkin Elmer Optima 4300 DV, Valencia, CA, USA). Three readings of aluminum concentration from each solution were recorded in parts per million. The amount of aluminum released per gram of cement (mg Al-/g cement) was calculated afterward.

### 2.5. Ethical Statement

Human dental pulp tissue collection performed in this study was approved by the Human Research Ethics Committee of the Faculty of Dentistry, Prince of Songkla University (No. of Approval: MOE 0521.1.03/998). For the use of pulp tissue samples, written informed consent was obtained from a human subject who participated.

### 2.6. Cell Culture

Pulp cells were cultured from normal human third molar from one adult patient aged about 18 years seen at the Dental Hospital, Faculty of Dentistry, Prince of Songkla University, with the approval of the Research Ethics Committee, Faculty of Dentistry, Prince of Songkla University (No. of Approval: MOE 0521.1.03/998). Primary culture of pulp cells was performed using an enzymatic method. Briefly, the pulp tissue was minced into pieces and digested in a solution of 3 mg/mL of collagenase Type I (Gibco, Invitrogen Corporation, Grand Island, NY, USA) and 4 mg/mL of dispase (Gibco, Invitrogen Corporation, Grand Island, NY, USA) for 1 h at 37 °C. After centrifugation, cells were cultured in alpha-modified Eagle’s medium (αMEM), supplemented with 20% FCS, 100 µM L-ascorbic acid 2-phosphate, 2 mM L-glutamate, 100 units/mL penicillin, and 100 µg/mL streptomycin, and incubated at 37 °C with 5% CO_2_. Pulp cells from passages 3 to 8 were used to test cytotoxicity in this study.

### 2.7. Cytotoxicity Assay

MTT assay was used to investigate the cytotoxicity of these RMGICs to pulp cells. There were 6 groups of specimens composed of different components, as shown in Table 1. Disc specimens with 5 mm diameter and 1 mm height were prepared using the same method described above, but the total amount of EGF was 40 ng per specimen for the groups with added EGF. There were also 2 groups, control and positive control, which were cells cultured in normal medium, and another group was cells cultured in medium with 10 ng/mL of EGF, respectively. Human dental pulp (HDP) cells were seeded at 1 × 10^5^ cells/well on the bottom compartment of a 12-well Transwell cluster plate (Costar; Corning Inc., Corning, NY, USA). Cells were fed with 1.5 mL of α-MEM supplemented with 10% FCS, 100 µM L-ascorbic acid 2-phosphate, 2 mM L-glutamate, 100 units/mL penicillin, and 100 µg/mL streptomycin, and incubated under 5% CO_2_ at 37 °C. After incubation for 24 h, the culture media was refreshed, and each specimen was placed on the upper compartment (Transwell insert, with 12 mm membrane diameter and 0.4 µm pore size), and 0.5 mL of the culture medium was added. Transwell plates and the culture medium allowed the released substances from the upper compartment to diffuse through the cells at the bottom compartment. Two MTT assays [21] were performed. The first assay was cells exposed to the specimens for 3 days. Another assay was performed after cells were exposed to specimens for 6 days. In the second assay, the media was refreshed after cells were exposed to the specimens for 3 days, and the experiment was continued until the sixth day of exposure. The experiments were repeated three times in each group.

### 2.8. Proliferative Assay

The effect of the released substances from the specimens on cell proliferation was investigated by counting cell numbers using a Coulter counter (Coulter^®^ Z^TM^, New York, NY, USA) after cells were exposed to the specimens for three periods each over the last two days. There were 6 groups of the specimens, the same groups as in the cytotoxicity test, and the control group was cells culture with normal media. HDP cells were seeded at 1.5 × 10^5^ cells/well on the bottom compartment of a 6-well Transwell cluster plate 24 h prior to the placement of the specimen with the same method as described above. The specimens were placed on the inserted part for two days before the specimen was moved to place on the new Transwell plate, which had already seeded the same number of cells 24 h before; the experiment was repeated for another two periods. After each period, cells were washed with PBS 2 times before trypsinization, and the cell number of each well was counted using the Coulter counter. The result was reported as percentages of cell numbers compared to the control of each time period, which was set as 100%.

### 2.9. Statistical Analysis

The logarithmic transformation of the EGF release plus one was applied to overcome the large variation of the EGF release and some zero results. ANOVA with repeated measures and the Tukey HSD hoc test [22] was used to analyze the transformed variable, Lg10 (EGF release+1). The releases of fluoride and aluminum from different formulas of modified RMGICs were compared using ANOVA with repeated measures and the Tukey HSD post hoc test. The results of MTT and proliferative assays were analyzed using a two-way analysis of variance (two-way ANOVA) and the Tukey HSD post hoc test with statistical significance set at *p* < 0.05.

## 3. Results

### 3.1. EGF Release

**Figure 1:**
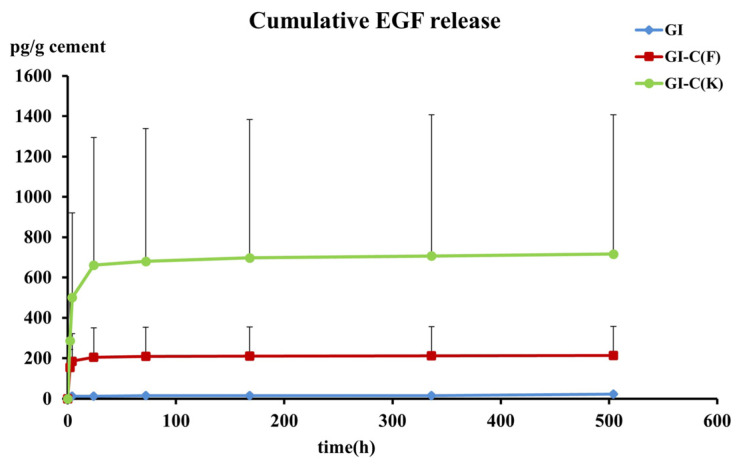
Cumulative release of EGF of different RMGICs with added EGF.

The result of the EGF release rate has high variation; the median and the value ranging from minimum to maximum of each group have been summarized in Table 2. The cumulative release of EGF has also been shown in Figure 1. By using logarithmic transformation, it was demonstrated that RMGIC modified by adding 15% of chitosan K and 5% of albumin supplemented with EGF, GI+C(K)+EGF, was the best group that gave a significantly higher release rate than the commercial RMGIC or GI group (*p* < 0.05, ANOVA with repeated measure and the Tukey HSD post hoc test). The specimens group GI+C(F) +EGF did not have any statistically significant difference in the EGF release rate compared to the other two groups. It was noted that the variation of all groups was high; however, the average released rate of EGF in the GI group was less than 5 pg/g of specimen per h in the first 2 and 4 h and reduced to 0 after 24 h, while the GI+C(K) group had a much higher release rate than 100 pg/g/h at the first 2 and 4 h like a burst release. After 24 h, the released rate dramatically reduced, but was still higher than 5 pg/g/h, and still released EGF in small, but detectable amounts for at least 3 weeks.

### 3.2. Fluoride and Aluminum Release

**Figure 2:**
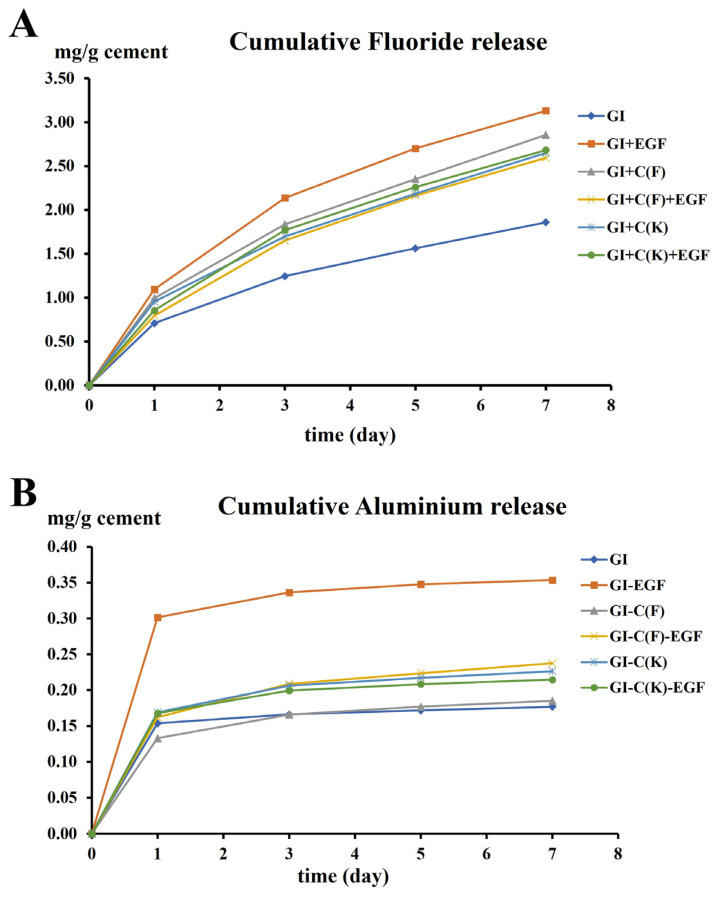
Cumulative release of fluoride in (**A**) and cumulative release of aluminum in (**B**). The GI+EGF group had the highest cumulative release of both fluoride and aluminum.

The results of fluoride and aluminum release have been presented as daily release rates, which are summarized in Table 3. The cumulative fluoride and aluminum release have been shown in Figure 2A and B, respectively. It was noticed that the release patterns of fluoride from all groups were not much different. Two phases of fluoride release were observed in all groups, an initial rapid fluoride washout phase (Day 1 to Day 3) followed by a slower steady elution of fluoride. The GI group gave the lowest average release rate, while the GI+EGF had the highest average release rate (*p* < 0.05). The average fluoride release rates of GI+C(F)+EGF, GI+C(K)+EGF, and GI+C(K) groups were not significantly different, but GI+C(F) had a significantly lower release rate than GI+C(F)+EGF.

The daily aluminum release from all RMGICs had the same pattern, which had the burst of release on the first day then reduced rapidly after 3 days and cannot be detected on day 5 in GI and GI+C(K) groups, while other groups also released at only about 0.01 mg/g/day. It was noticed that the GI+EGF had the significantly highest aluminum release rate (*p* < 0.05), while other groups had no statistically significant difference (*p* > 0.05).

### 3.3. Cytotoxicity Assay

**Figure 3:**
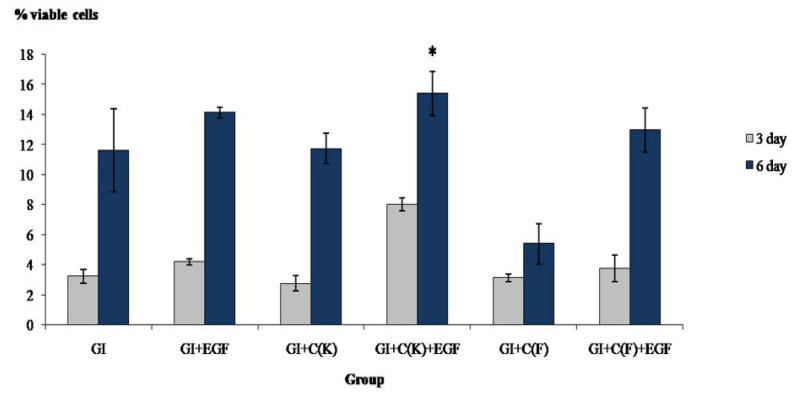
MTT assay of HDP cells exposed to the specimens for 3 and 6 days. * indicated significant (*p* < 0.05).

The cytotoxicity of the specimens was investigated at two time intervals, 3 and 6 days, with an MTT assay, as shown in Figure 3. The result was analyzed with 2-way ANOVA, and the Tukey post hoc test demonstrated that the GI+C(K)+EGF group had the highest percentages of cell viability (*p* < 0.05). Cells exposed to the specimens for 6 days had significantly higher percentages of survival cells than 3 days in all groups, which means that the toxicity of the specimens reduced significantly when exposing cells for longer than 3 days. The positive control groups that were cells cultured in media supplemented with 10 ng/mL of EGF for 3 and 6 days had percentages of cell viability at 109.5 ± 14.8 and 101.3 ± 2.2 mg/g cement, respectively.

### 3.4. Proliferative Assay

**Figure 4:**
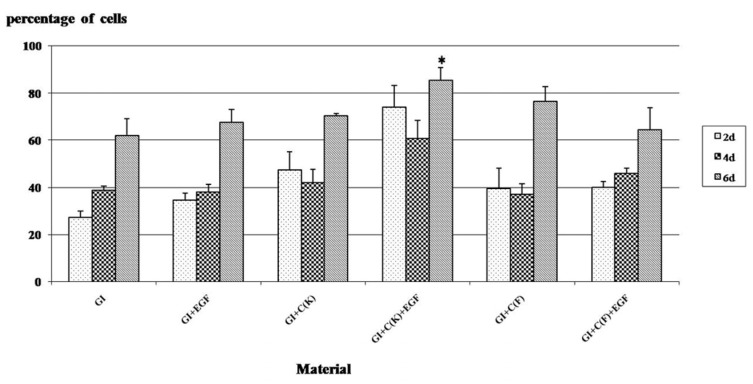
Percentages of cell number using Coulter counter after cells were cultured with different RMGICs for three periods. * indicated significant (*p* < 0.05).

Figure 4 revealed the results of percentages of cell number after cells were cultured with different RMGICs at three time periods. The specimens in group GI+C(K)+EGF had the highest average percentages of cell numbers (*p* < 0.05), and the third incubation (6 d) of the specimens gave a significantly higher (*p* < 0.05) percentage of cell numbers than the first (2 d) and the second (4 d), which may demonstrate the promotion of prolonged cell proliferation of the GI+C(K)+EGF group for at least up to 6 days.

## 4. Discussion

This study attempted to develop a glass-ionomer cement, which is able to control the release of bioactive molecules and also maintain the important properties of the glass-ionomer cement. These are particular to fluoride and aluminum release. Aluminum should be released only in a short period of time with a non-toxic level. The percentages of chitosan and albumin used in this study were the result of several prior studies, which had been performed by adjusting the percentages of chitosan and albumin in order to obtain a satisfactory prolonged release rate.

The pattern of the EGF release could be observed in the modified RMGICs. The GI-C(K)+EGF group appeared clearly composed of two phases. The first phase is the high release rate of the burst effect, whereas EGF can be detected in high concentrations at 2 and 4 h after immersion of the specimens. In the second phase (after 24 h), the amount of EGF was dramatically reduced, but could still be detected until 3 weeks. This result was similar to our prior study that reported that chitosan-modified glass-ionomer cement was able to prolong the release of protein [7]. Moreover, this investigation here also showed that the molecular weight of chitosan influenced releasing property of the cement. The results suggested that RMGIC modified by adding chitosan K at 15% and albumin at 5% by weight released a high amount of EGF and can prolong the release for at least 3 weeks. Chitosan can prolong protein release, possibly due to the formation of a polyelectrolyte complex between the cationic group of chitosan and the anionic group of poly (acrylic acid) [23,24]. Albumin was added to prolong the release of growth factor [25]. Bovine serum albumin was added to RMGIC due to its action as a carrier protein [8]. It can help the release of small proteins like EGF without any cytotoxic to HDP cells. It was concerned that EGF release from this chitosan-modified RMGIC still showed some variation amount of EGF release. The degradation of this small protein (EGF) or the poor distribution of this growth factor in the specimens should be the focus of further investigation.

The results of cytotoxicity (MTT assay) from Figure 3 revealed that the GI+C(K)+EGF group had the least cytotoxicity. Moreover, this group released substances that can promote the growth of pulp cells (Figure 4) compared to the control groups for at least 6 days, which may be referred to as the prolonged release of EGF, as demonstrated in Table 2. This group still released a detectable amount of EGF after 7 days and had its bioactivity in promoting cell growth.

The result clearly showed that all the chitosan-modified RMGICs have the same fluoride release pattern as general RMGIC. They all released fluoride at the highest rate on the first day as a burst effect and reduced rapidly after 2, 3, and 5 days and started to be slowly released, but continuously thereafter. Two mechanisms have been proposed for fluoride release from glass ionomers into an aqueous environment. One mechanism is a short-term reaction, which involves rapid dissolution from the outer surface into solution, whereas the second step is more gradual and results in the sustained diffusion of species through the bulk material [26,27]. It has been found that after the initial burst effect, resin-modified glass-ionomer cement continues to release small amounts of fluoride in vitro for 1-2.7 years [26,28].

Aluminum release of these different RMGICs had the highest rate as a burst effect on the first day, then rapidly reduced. It could not be detected at the end period of seven days. It was noticed that the GI+EGF group released the highest amount of average fluoride and aluminum, especially on the first day. This may be because the EGF protein might influence the setting reaction or polymerization of RMGIC. Considering the safety of the released aluminum [29], it was reported that the current recommended level of the US Department of Agriculture for aluminum’s maximum permissible dose per day is 0.10–0.12 mg Al/kg/day [30,31,32]. The recommended amount is much higher than the released aluminum from the modified RMGIC used here in this study. It was found that a single glass-ionomer filling would provide roughly 0.5% of the maximum permissible dose per day, and it was believed that there should be no adverse health effects from glass-ionomer fillings [33]. In addition, a study showed that the release of aluminum from glass-ionomer material is able to assist the biological effect of fluoride against the acidogenicity of *S. mutans* biofilms [34]. In this study, chitosan-modified RMGICs groups release greater rates of fluoride than control RMGIC, the same result as a recent study, which reported that the inclusion of chitosan in RMGIC increased fluoride release [35]. Chitosan also enhanced fluoride release in conventional GIC [16,36].

It was interesting that the GI+C(K)+EGF group, where the C(K) had lower molecular weight with a higher degree of deacetylation, seems to give better results in EGF release and, in particular, had significantly lower cytotoxicity and could promote cell proliferation. This corresponded with the studies reporting that chitosan with low molecular weight and a high degree of deacetylation was suitable for drug delivery [37,38]. Chitosan-based polyelectrolyte complexes have been broadly considered for drug delivery systems, which include the polyelectrolyte complexes formed by chitosan and poly (acrylic) acid [12]. This modified glass ionomer cement revealed the potential property of prolonged release of bioactive molecules. However, this study is a preliminary in vitro experiment, which has limitations in interpretation. Further investigations will be required to examine such aspects as its chemical, physical and mechanical properties, as well as the proper type and amount of bioactive molecules that should be added for specific therapeutic purposes.

## 5. Conclusions

This study demonstrated that chitosan resin-modified glass ionomer cement added with EGF could prolong the release of EGF; even though the amount released was very small after 72 h, it still retained its bioactivity. This modified RMGIC also had the property of prolonging fluoride release and the release of a limited amount of aluminum. The capability to control the release of bioactive molecules in this biomaterial may assist in regenerating pulp–dentin complexes.

## Figures and Tables

**Table 1:** Powder compositions for various groups of RMGICs specimens.

Group of Specimens	Powder Compositions
GI	Fluoroaluminosilicate glass.
GI+EGF	Fluoroaluminosilicate glass with added EGF during mixing the cement.
GI+C(F)	Fluoroaluminosilicate glass, 15% chitosan Mw 545 kDa, and 5% BSA.
GI+C(F)+EGF	Fluoroaluminosilicate glass, 15% chitosan Mw 545 kDa, and 5% BSA with added EGF during cement mixing.
GI+C(K)	Fluoroaluminosilicate glass, 15% chitosan Mw 62 kDa, and 5% BSA.
GI+C(K)+EGF	Fluoroaluminosilicate glass, 15% chitosan Mw 62 kDa, and 5% BSA with added EGF during cement mixing.

**Table 2:** Median and (minimum–maximum) rate of EGF release (pg/g cement/h) from a different formula of RMGICs with added EGF at seven time periods.

Time (h)	Type of Material		
	GI+EGF ^a^	GI-C(F)+EGF ^a,b^	GI-C(K)+EGF ^b^
2	3.362	131	253.31
	(0.00–6.38)	(50.06–277.62)	(31.44–529.67)
4	3.865	2.788	70.296
	(0.00–13.56)	(0.91–74.44)	(5.75–238.50)
24	0	0.773	1.71
	(0.00–0.01)	(0.16–2.42)	(0.27–23.82)
72	0	0.03	0
	(0.00–0.38)	(0.00–0.31)	(0.00–1.68)
168	0	0.004	0
	(0.00–0.00)	(0.00–0.06)	(0.00–0.61)
336	0	0.001	0.001
	(0.00–0.00)	(0–0.01)1	(0.00–0.02)
504	0.008	0.01	0.001
	(0.00–0.22)	(0.00–0.05)	(0.00–0.33)

^a,b^ Different letters are statistically significantly different at *p* < 0.05.

**Table 3:** Fluoride and aluminum release rates (mg/g cement/day) from various formulations of glass-ionomer cement over a period of 7 days presented as means (SD).

	Day1	Day3	Day5	Day7
	Rate of fluoride release (mg/g cement/day)			
**GI ^a^**	0.71 (0.08)	0.25 (0.03)	0.15 (0.00)	0.14 (0.01)
**GI+EGF ^d^**	1.09 (0.08)	0.53 (0.02)	0.29 (0.01)	0.22 (0.01)
**GI+C(F) ^c^**	0.99 (0.07)	0.41 (0.02)	0.25 (0.01)	0.24 (0.01)
**GI+C(F)+EGF ^b^**	0.79 (0.03)	0.42 (0.02)	0.25 (0.01)	0.21 (0.01)
**GI+C(K) ^b,c^**	0.95 (0.08)	0.37 (0.02)	0.24 (0.02)	0.23 (0.01)
**GI+C(K)+EGF ^b,c^**	0.86 (0.11)	0.46 (0.06)	0.25 (0.01)	0.21 (0.01)
	**Rate of aluminum release (mg/g cement/day)**			
**GI ^a^**	0.15 (0.01)	0.01 (0.00)	0.00 (0.00)	0.00 (0.00)
**GI+EGF ^b^**	0.30 (0.01)	0.02 (0.00)	0.01 (0.00)	0.00 (0.00)
**GI+C(F) ^a^**	0.16 (0.02)	0.02 (0.00)	0.01 (0.00)	0.01 (0.00)
**GI+C(F)+EGF ^a^**	0.13 (0.01)	0.02 (0.00)	0.01 (0.00)	0.00 (0.00)
**GI+C(K) ^a^**	0.17 (0.02)	0.02 (0.00)	0.01 (0.00)	0.00 (0.00)
**GI+C(K)+EGF ^a^**	0.17 (0.04)	0.02 (0.00)	0.00 (0.00)	0.00 (0.00)

^a,b,c^ Different letters are statistically significantly different at *p* < 0.05.

## Data Availability

Data will be available on request.
